# Successful surgical case of refractory chronic expanding intrapericardial hematoma treated with preoperative coil embolization of the feeding vessels

**DOI:** 10.1002/ccr3.2252

**Published:** 2019-06-28

**Authors:** Masaro Nakae, Hiroki Mizoguchi, Masao Yoshitatsu, Koichi Toda, Yoshiki Sawa

**Affiliations:** ^1^ Department of Cardiovascular Surgery Kansai Rosai Hospital Amagasaki Japan; ^2^ Department of Cardiovascular Surgery Osaka University Graduate School of Medicine Suita Japan

**Keywords:** chronic expanding intrapericardial hematoma, hybrid therapy, preoperative coiling, recurrent hematoma

## Abstract

Complete surgical resection of chronic expanding intrapericardial hematoma was often difficult because of the severe adhesion. Preoperative coil embolization of the feeding vessels can prevent recurrent expanding of the residual hematoma and would achieve good results.

## 1 INTRODUCTION

Chronic expanding intrapericardial hematoma is rare that develops after surgery, trauma, or epicardial injury.^1^ A hematoma resolves rapidly without clinical problems; however, chronic expanding hematomas persist and slowly expand for more than 1 month after the initial injury, and lead to the formation of a capsule around the hematoma with novel feeding vessels, resulted in growing to the space‐occupying lesion. We describe a successful surgical case of refractory chronic expanding intrapericardial hematoma after mitral valve surgery treated with preoperative coil embolization of the feeding vessels.

## 2 PATIENT PROFILE

**Figure 1:**
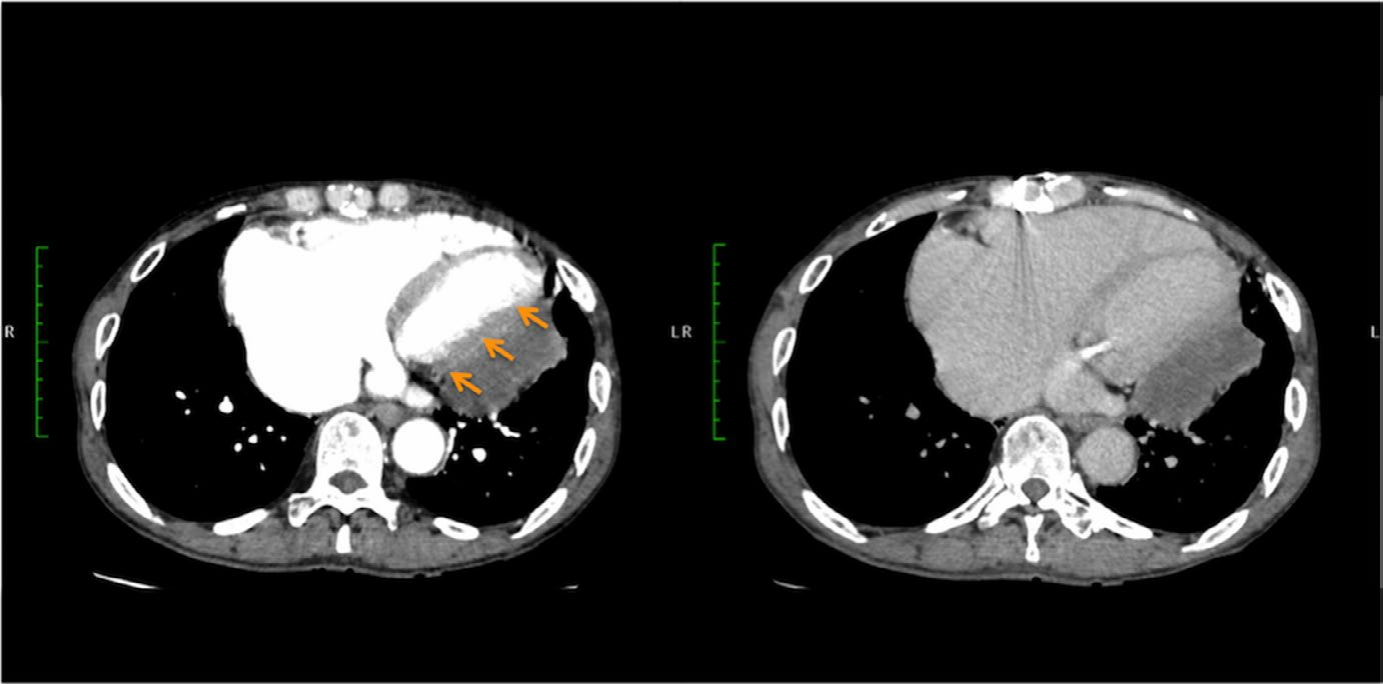
Preoperative contrast‐enhanced computed tomography showing the intrapericardial hematoma compressing the left ventricle. The hematoma was enhanced in the delayed‐phase imaging

**Figure 2:**
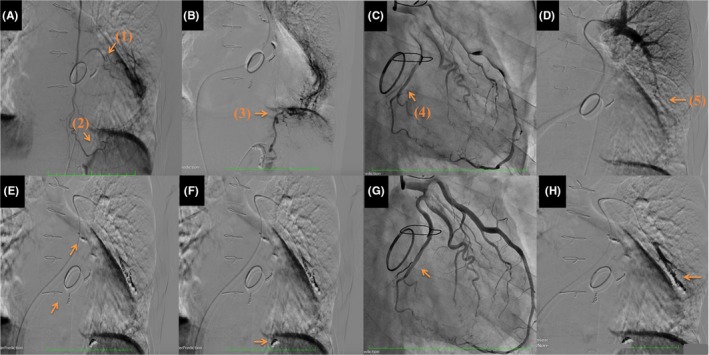
A, B, C, D, Preoperative angiography showing the feeding vessels of the chronic expanding intrapericardial hematoma. (1) Left intercostal artery, (2) left musculophrenic artery, (3) left infraphrenic artery, (4) left circumflex branch of left coronary artery, and (5) left pulmonary artery. E, F, G, H, Each vessel was embolized preoperatively

**Figure 3:**
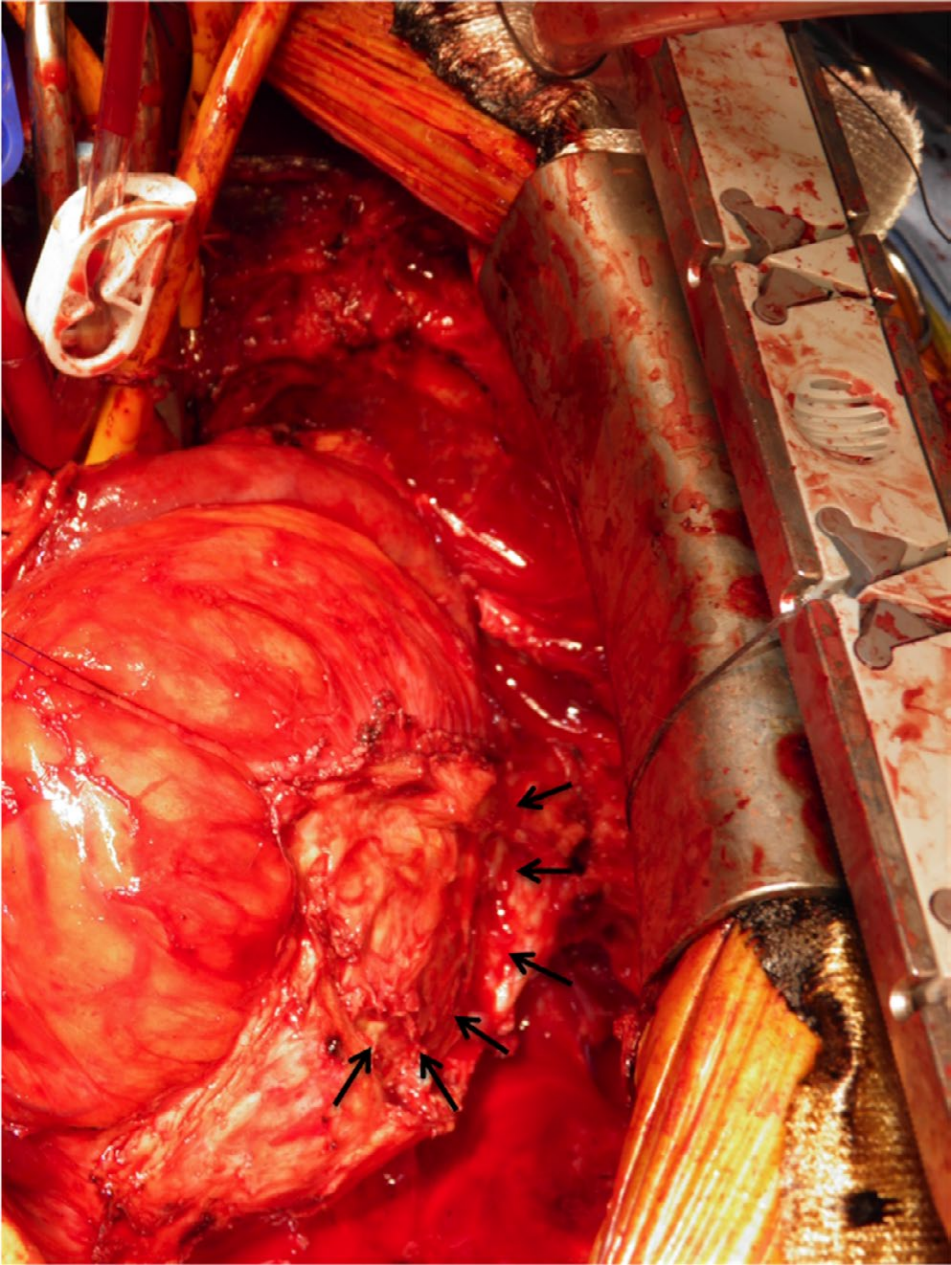
Intraoperative findings. After the pericardium was opened, the extent of the hematoma and the thickened epicardium were revealed (shown by arrows). The thick pericardium was severely adhered to the myocardium

A 68‐year‐old man presented with leg edema and dyspnea on effort. He had a history of mitral valve plasty and left atrial maze procedure 8 years ago. Three years after the surgery, follow‐up transthoracic echocardiography (TTE) and chest computed tomography (CT) demonstrated a space‐occupying lesion close to posterolateral wall of the heart then left ventricular (LV) diastolic function decreased. Despite medication, LV diastolic function gradually worsened and resulted in pulmonary hypertension and functional tricuspid regurgitation (TR); therefore, a resection of the hematoma was performed via a left thoracotomy. Histopathological diagnosis was intrapericardial hematoma. A complete resection was not feasible, hence, 2 years after the procedure, follow‐up TTE and chest CT demonstrated relapsing intrapericardial hematoma in situ, and furthermore, the hematoma has gradually expanded (Figure 1). The LV diastolic dysfunction due to compression of hematoma and right‐sided heart failure due to functional TR has worsened to severe, and the laboratory data showed the elevation of brain natriuretic peptide score and the decreasing platelets resulting from progression of liver dysfunction. Then, the surgical intervention was planned for refractory chronic expanding hematoma. Preoperative contrast‐enhanced chest CT showed that the hematoma was enhanced in delayed‐phase imaging; then, we might suspect that some feeding artery to the hematoma has existed. Prior to the surgical intervention, selective angiographies were performed to identify the feeding vessels. An angiography showed multiple feeding vessels from circumflex branch of the left coronary artery, left intercostal artery, left inferior phrenic artery, and left pulmonary artery (Figure 2). To reduce the intraoperative bleeding, coil embolization of the feeding vessels was performed prior to the surgical intervention. Postcoil embolization electrocardiogram showed no ischemic change. Through a median sternotomy under cardiac arrest, at first, we performed tricuspid annuloplasty by using 30‐mm Carpentier‐Edwards Physio tricuspid annuloplasty ring (Edwards Lifesciences). Secondly, we exposed the encapsulated hematoma located posterolateral of the LV wall, in which adhering to the myocardium and the surrounding tissues, and then, the thick pericardium was opened. The capsule was filled with partially organized hematoma, and it was easily removed but the thick pericardium was severely adhered to the myocardium; therefore, we decided that a complete resection of the pericardium was impracticable. Obvious feeding vessels were ligated (Figure 3).

Postoperative TTE demonstrated recovery of LV cardiac function, and the LV wall compression was disappeared. Contrast‐enhanced chest CT and angiography showed reduction in space‐occupying lesion and no residual feeding vessels. Postoperative course was uneventful, and he discharged on day 26 after operation.

## 3 DISCUSSION

Chronic expanding hematoma was first reported by Reid et al,^2^ developing in various locations for more than a month after the initial bleeding. In pericardial cavity, a few cases were also reported after surgery, trauma, or epicardial injury^1^, ^3^ and the cause of the chronic expanding hematoma remains unclear. Labadie et al^4^ have suggested that the inflammation caused by the initial event induces bleeding from fragile capillaries into the pericardium resulted in forming a fibrous capsule. Therefore, a complete resection, including the capsule, is the preferred surgical procedure, as incomplete resection might result in a recurrence of the hematoma.^4^, ^5^ However, the adhesion around the capsule of the hematoma is predicted severe, hence, the complete removal of the capsule is extremely difficult, and this issue is controversial.

Recently, the utility of preoperative coil embolization for the feeding vessels to the chronic expanding hematoma has been reported.^5^, ^6^ The gradual formation of a hematoma accompanies leakage of blood from the new vessels, which increases permeability through a plasminogen activator that is precipitated in the clot.^4^ An essence of this disease is blood supply to the hematoma; therefore, the coil embolization of feeding vessels might prevent perioperative bleeding and recurrence.

In our case, the capsule of the hematoma was formed by the epicardium and severely adhered to the myocardium; therefore, a complete resection of the pericardium was impracticable. Moreover, neovascularization and blood supply from several feeding vessels might induce the gradual expansion of the capsule; hence, we thought that the preoperative identification of the feeding vessels and severance of blood supply by coil embolization seems to be useful for the prevention of the recurrence.

In conclusion, we reported the refractory case of chronic expanding intrapericardial hematoma treated with preoperative coil embolization of the feeding vessels. In cases where the resection of the capsule is difficult, we thought that this method would be very useful and may contribute to the prevention of the recurrence.

## CONFLICT OF INTEREST

None declared.

## AUTHOR CONTRIBUTION

MN: treated the patient and drafted the manuscript. HM: supervised the manuscript. MY: was responsible for the case. KT and YS: supervised the surgical strategy of the case.

## References

[1] Kainuma S , Masai T , Yamauchi T , et al. Chronic expanding intrapericardial hematoma after coronary artery bypass surgery presenting with congestive heart failure. Ann Thorac Cardiovasc Surg. 2008;14:52‐54. 18292743

[2] Ried JD , Kommareddi S , Lankerani M , Park MC . Chronic expanding hematomas. A clinicopathologic entity. JAMA. 1980;244:2441‐2442. 6448929

[3] Sughiura T , Nishida H , Ishitoya H , et al. Chronic expanding intrapericardial hematoma after pericardial paracentesis. J Card Surg. 2006;21:491‐493. 16948767 10.1111/j.1540-8191.2006.00307.x

[4] Labadie EL , Glover D . Physiopathogenesis of subdural hematomas. Part 1: histological and biochemical comparisons of subcutaneous hematoma in rats with subdural hematoma in man. J Neurosurg. 1976;45:382‐392. 956874 10.3171/jns.1976.45.4.0382

[5] Kuwata T , Uramoto H , Tanaka F . Preoperative embolization followed by palliative therapy for patients unable to undergo the complete removal of a chronic expanding hematoma–a case report. Asian J Surg. 2013;36:40‐42. 23270824 10.1016/j.asjsur.2012.06.006

[6] Kuriyama M , Mitsukawa N . Preoperative arterial embolizations of a huge, chronic, expanding hematoma to inhibit intra‐operative massive bleeding: a case report. J Plast Reconstr Aesthet Surg. 2009;62(7):e203‐e205. 19369128 10.1016/j.bjps.2009.01.072

